# A retrospective study correlating sonographic features of thyroid nodules with fine-needle aspiration cytology in a South African setting

**DOI:** 10.4102/sajr.v23i1.1749

**Published:** 2019-06-26

**Authors:** Mark A. Nicolaou, Kathleen Jacobs, Sindeep Bhana, Kershlin Naidu, Veronique Nicolaou

**Affiliations:** 1 Department of Diagnostic Radiology, University of the Witwatersrand, Johannesburg, South Africa; 2 Department of Diagnostic Radiology, Chris Hani Baragwanath Hospital, Johannesburg, South Africa; 3 Department of Endocrinology, Chris Hani Baragwanath Hospital, Johannesburg, South Africa; 4 Endocrinologist, Netcare Waterfall City Hospital, Johannesburg, South Africa; 5 Department of Endocrinology, Chris Hani Baragwanath Hospital, Johannesburg, South Africa

**Keywords:** Thyroid, nodule, fine-needle aspiration, FNA, American Thyroid Association, ATA, thyroid cancer

## Abstract

**Background:**

Thyroid nodules are prevalent worldwide. Detection rates are increasing because of the use of ultrasonography. Ultrasound has become the first-choice imaging modality in evaluating nodules. The decision to perform an US-guided fine-needle aspiration (FNA) is based on a nodule’s sonographic features. Thus, it is essential to accurately risk stratify thyroid nodules so that they are appropriately referred for FNA.

**Objectives:**

The aim of this study was to correlate the ultrasound imaging features of thyroid nodules with FNA cytology and surgical histopathology results, and to risk stratify patients using the American Thyroid Association (ATA) classification for each imaging characteristic with the likelihood of the nodule being malignant.

**Method:**

Retrospective analysis of a thyroid ultrasound database at Chris Hani Baragwanath Academic Hospital, over the period 2015–2017. Frequencies and percentages were used to summarise the data. Univariate logistic regression analyses were used to assess the accuracy of sonographic features in predicting the histologically determined diagnosis for thyroid tumours.

**Results:**

A total of 113 nodules underwent FNA, of which 104 were diagnostic. The best three ultrasound features that pose a higher risk for malignancy are absent halo, presence of microcalcifications and hypoechoic appearance. No single nodule feature is an absolute indicator for malignancy. There is a high agreement between ATA classification and cytopathology or histology when nodule features are grouped into clusters. Agreement between the ATA classification and cytopathology/histology was 86.7% with a kappa of 0.714. The agreement between the cytopathology FNA results and lobectomy histopathology was 98.8% with a kappa of 0.973.

**Conclusion:**

This study contributes to the paucity of data available for sub-Saharan Africa and provides reassurance that our results are consistent with international studies. The study confirms that the usage of a thyroid nodule classification system improves characterisation and increases accuracy in detecting thyroid malignancies, thus sparing many patients the morbidity of unnecessary thyroid surgery.

## Introduction

### Background

Thyroid nodules are prevalent worldwide and seen in patients of all age groups. Detection rates are higher than previously reported with the ever-increasing use of thyroid ultrasonography. Several large international studies report that up to 76% of women have at least one thyroid nodule and as many as 57% of routine autopsies discover incidental nodules.^1^ The majority of nodules are asymptomatic and are benign in 80%–92% of cases.^2^ When a thyroid nodule is discovered incidentally or clinically, there is a necessity for workup and further investigation.

### Thyroid nodules and ultrasound

A thyroid nodule is defined as a distinct focal lesion within the thyroid parenchyma.^3^ Ultrasound has become the first-choice imaging modality in screening and detection of thyroid nodules. This is not only because of its excellent spatial resolution compared to computed tomography (CT) and magnetic resonance imaging (MRI), but also because the thyroid gland is easily visualised sonographically because of its superficial location anatomically.^2^ The next step in the evaluation of a thyroid nodule is to perform an ultrasound-guided fine-needle aspiration (FNA) if the nodule/s meet certain sonographic criteria. Thyroid scintigraphy is performed in the evaluation of thyroid nodules in patients with biochemical hyperthyroidism.

There is a relatively low risk of nodules being malignant with only 4%–8% of thyroid nodules diagnosed as malignant on cytology.^2,4^ Despite this low risk, the discovery of a nodule on ultrasound can lead to anxiety for the patient and may lead to unnecessary FNA and/or surgery being performed. Clear sonographic guidelines are essential in determining whether a FNA is indicated because sonography alone cannot definitively diagnose a malignancy but may obviate the need for FNA.^5^ Since the introduction of ultrasound-guided FNA, there has been a significant increase in the likelihood of malignancy at surgical resection.^6^

### Thyroid nodule classification systems

There are numerous thyroid nodule risk stratification systems that have been developed. The two most commonly used systems are the American Thyroid Association (ATA) management guidelines and the American College of Radiology Thyroid Imaging Reporting and Data System (ACR TIRADS.).^7^

Both systems combine thyroid nodule ultrasound findings and give an estimated risk of malignancy.^8,9^ They both utilise ultrasound features of the thyroid nodule that include

- echogenicity of the nodule
- sharpness of the border/margin of the nodule
- shape of the nodule (taller-than-wide configuration in transverse plane)
- presence/absence of a halo around the nodule
- microcalcifications
- internal architecture or composition of the nodule (spongiform, cystic or solid)
- nodule vascularity (peripheral vs. central vascularity).

The difference between the two aforementioned systems is that the ATA guidelines use a pattern-based approach rather than a scoring system utilised in the ACR TIRADS.^6,9,10^

### Cytopathological evaluation

Once an FNA is performed, the sample is evaluated and reported on by a cytologist. In order for the cytopathological report to be universal and unambiguous, a standardised format of reporting was developed in the form of the Bethesda System for Reporting Thyroid Cytopathology.^11^

This system classifies results into six diagnostic categories:

1. non-diagnostic or unsatisfactory
2. benign
3. atypia of undetermined significance (AUS) or follicular lesion of undetermined significance (FLUS)
4. follicular neoplasm or suspicious for a follicular neoplasm
5. suspicious for malignancy
6. malignant

The six categories stratify the risk associated with malignancy (Table 1)^11^.

**TABLE 1:** Risk stratification for categories of the Bethesda system for reporting thyroid cytopathology.^6^

Diagnostic category	Description	Risk of malignancy (%)
**I**	Non-diagnostic/unsatisfactory	1–4
**II**	Benign	0–3
**III**	Atypia or follicular lesion of undetermined significance	5–15
**IV**	Follicular neoplasm or suspicious for follicular neoplasm	15–30
**V**	Suspicious for malignancy	60–75
**VI**	Malignant	97–99

*Source*: Cibas ES, Ali SZ. The 2017 Bethesda system for reporting thyroid cytopathology. J Am Soc Cytopathol. 2017;6:217–222. https://doi.org/10.1016/j.jasc.2017.09.002

The aim of this study was to evaluate the predictive value of ultrasonographic findings in the diagnosis of benign and malignant thyroid nodules in our setting and, furthermore, to confirm the accuracy of the cytopathological diagnosis where histopathological results were available.

## Research methods and design

This retrospective cross-sectional study was performed at the Chris Hani Baragwanath Academic Hospital (CHBAH) in Soweto, Johannesburg. Patients seen at various outlying clinics and outpatient departments in CHBAH were referred for ultrasound-guided thyroid FNAs at the CHBAH Endocrine Clinic.

Adult patients with normal thyroid function tests were included in the study if the FNAs were performed at the CHBAH Endocrinology Clinic. Those with deranged thyroid functions were only included if they were biochemically hypothyroid. Patients were excluded from the study if the FNA cytology results were non-diagnostic/unsatisfactory according to Bethesda classification or if they initially had AUS/FLUS cytology and they did not return for a repeat FNA – that is, lost to follow-up.

### Ultrasound examination and fine-needle aspiration technique

Two dedicated endocrinologists trained in thyroid sonography performed the ultrasound-guided FNAs, thus reducing inter-user variability. Because only two endocrinologists were trained in thyroid ultrasonography and thyroid FNA, all results from the clinic were derived from their findings. The thyroid ultrasound was performed and if indicated, based on imaging features, an FNA was performed at the same time. In cases of multiple thyroid nodules, the most suspicious nodule, based on imaging features, was sampled.

The endocrine unit uses a Siemens ACUSON X300 machine with a linear probe frequency range of 5 MHz–14 MHz and a GE Logiq P5 with a linear probe frequency range of 5 MHz–13 MHz. Fine-needle aspirations were performed by the two endocrinologists using a standardised international thyroid FNA technique, namely, the patient in supine position with the neck hyper-extended. A BD PrecisionGlide 27G (1¼ inch) needle attached to a plastic 10 mL syringe was used to aspirate the nodule under direct ultrasound guidance. The needle was passed into the nodule for a duration of 10–30 s with no negative pressure applied to the syringe. Two to three needle passes were obtained from a nodule. No local anaesthesia was used.

Each needle specimen was smeared on four to eight glass slides. Half the slides were left to air dry and the remainder of the slides were prepared with 95% ethyl alcohol cytological fixative spray. The glass slides were then submitted to the National Health Laboratory Services (NHLS) for cytological evaluation by a single cytopathologist, which reduced inter-observer variability.

### Data collection

A database of 113 thyroid FNAs was constructed using records from the Endocrine clinic dating from May 2015 until September 2017. Data were recorded in a spreadsheet using Microsoft Excel. Data were excluded where cytopathological results were reported as non-diagnostic/unsatisfactory for evaluation and those patients who were lost to follow-up after their initial FNA cytopathology demonstrated AUS/FLUS.

The demographic data of the patients were anonymised and included the age and gender. The following thyroid nodule characteristics on ultrasound were recorded: transverse nodule size, presence of microcalcifications, internal nodule vascularity on colour Doppler, echogenicity relative to neck strap muscle echogenicity, solid nodule architecture, taller-than-wide configuration (transverse orientation) and presence or absence of a halo. The ATA classification system was used to categorise the nodules.

The cytopathology result of all the FNAs was followed up and recorded according to the Bethesda classification system. Patients were referred for surgery if their FNA results were Bethesda III/IV (two abnormal FNA results), Bethesda V (suspicious for malignancy) or Bethesda VI (malignant). Histological results of these patients were also included in the database.

### Data analysis

Frequencies and percentages were used to summarise the data. Univariate logistic regression analyses were used to assess the accuracy of the sonographic features in predicting the histologically determined diagnosis for thyroid tumours. Positive predictive values, negative predictive values, sensitivity and specificity were determined from the model and plotted as a bar graph for each sonographic feature. Agreement between ATA classification, cytopathology and histology results, as well as cytopathology and histology results, was determined using Cohen’s kappa. All analyses were performed at the 95% confidence interval. The data were analysed using Stata version 13.1 (StatCorp, USA).

## Ethical consideration

The Human Research Ethics Committee at the University of the Witwatersrand granted ethics clearance on 04 October 2017. Clearance Certificate number is M170970. Consent obtained from CHBAH Medical Advisory Committee.

## Results

A final total of 100 thyroid nodule FNAs were included in the study of 113 patients. Only 13 thyroid nodule FNAs were excluded from the study, 9 of which for being non-diagnostic/unsatisfactory and a further 4 patients who were lost to follow-up after initially having AUS/FLUS on their first FNA cytopathology. There were 84 females and 16 males in the study. The age range was 16–82 years. The nodule size range was 10 mm–60 mm with a mean nodule size of 30.5 mm.

Forty-two of the 100 patients were referred for surgical intervention; this was according to the patients’ Bethesda classification or if they had repeat AUS/FLUS on cytology results. Of all the patients referred for surgery, 79% were confirmed malignant on histopathology. Of the 100 thyroid nodules, 67% were benign and 33% were malignant on histopathology.

**FIGURE 1:**
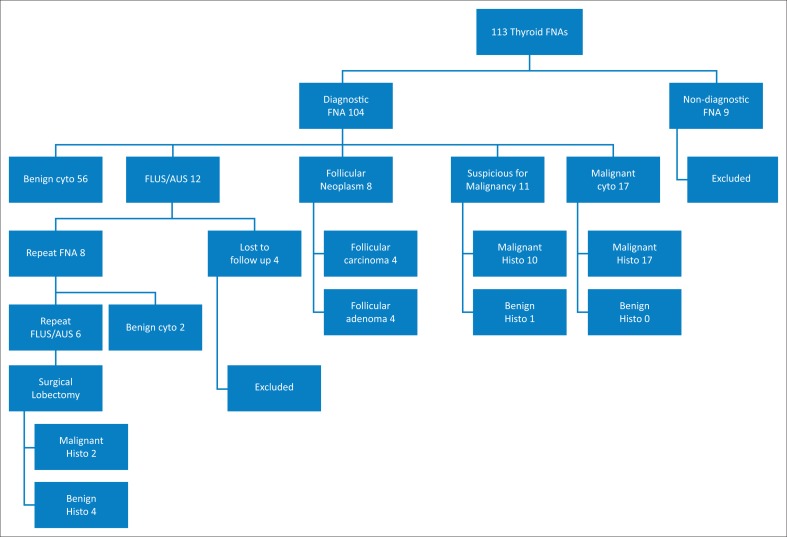
Summary of results of thyroid nodules sampled.

Half of the patients referred for surgery because of follicular neoplasm on cytopathology had follicular carcinoma on histopathology. Patients who had repeat AUS/FLUS were also referred for surgery – 25% of these patients had malignant histology. There was only one patient who had cytopathology suspicious for a Hurthlë cell neoplasm who had benign histopathology. The number of FNAs and outcomes of the cytopathology and histology results are graphically presented in Figure 1.

**FIGURE 2:**
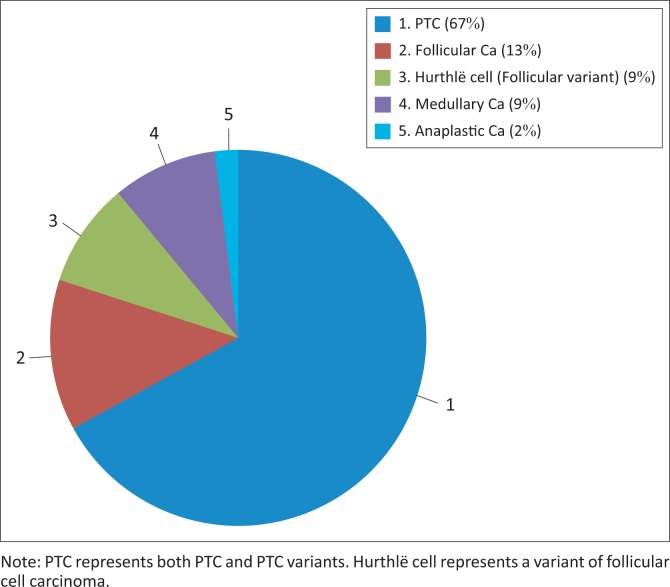
Breakdown of malignant nodules with histology.

The histology results determined the following: 17 papillary thyroid carcinoma (PTC) and five follicular variant of PTC (67%), four follicular carcinomas (13%), three Hurthlë cell carcinomas (follicular cell variant) (9%), three medullary carcinomas (3%) and one anaplastic carcinoma (2%). Nine of the surgical specimen histology results were benign. These results are presented in Figure 2.

The diagnostic performance of each thyroid nodule feature on sonar is outlined in Table 2 demonstrating the sensitivity, specificity, positive predictive value (PPV) and negative predictive value (NPV).

**TABLE 2:** Diagnostic performance of each thyroid nodule characteristics in detecting malignancy.

Feature	Sensitivity (%)	Specificity (%)	PPV	NPV	Positive LHR
Taller-than-wide	12	95.5	57	68.8	2.7
Absent halo	36.4	95.5	80	75.3	8.1
Hypoechoic	54.5	89.5	72	80	5.2
Solid	43.7	77.0	63	59.7	1.9
Microcalcifications	27.3	91.1	60	71.7	3.1
Vascularity	41.6	76.2	30	79	1.7
Size >1 cm	3.0	98.5	50	67.3	2.0

PPV, positive predictive value; NPV, negative predictive value; LHR, likelihood ratio.

Agreement between the ATA classification and cytopathology/histology was 86.7% with a kappa of 0.714 (see Table 3). The agreement between the cytopathology FNA results and surgical histopathology was 98.8% with a kappa of 0.973.

**TABLE 3:** Agreement table between American Thyroid Association classification and cytology/histological outcomes.

ATA classification	Cytology/histology outcome				
	Nodules	Benign		Malignant	
		*n*	%	*n*	%
Very low suspicion	10	9	90	1	10
Low suspicion	47	43	91	4	9
Intermediate suspicion	23	12	52	11	48
High suspicion	20	3	15	17	85

ATA, American Thyroid Association.

## Discussion

In this retrospective study, we found that the best three ultrasound features that pose a higher risk of malignancy, using a positive likelihood ratio, are nodules with an absent halo, the presence of microcalcifications and those with a hypoechoic appearance. However, no single feature is an absolute indicator for malignancy as none had a clinically relevant positive likelihood ratio of greater than 10.

These results reflect findings similar to that of the international literature. A systematic review and meta-analysis of thyroid ultrasound features by Remonti et al.^5^ showed comparable outcomes regarding prediction of malignancy by specific nodule features. In our study group, both microcalcifications (91.1%) and taller-than-wide configuration (95.5%) were found to be highly specific in malignant nodules. This was also noted by Remonti et al.^5^ with microcalcifications and taller-than-wide configuration having a specificity of 87.8% and 96.6%, respectively.

The aforementioned meta-analysis also found that single ultrasound features had a generally low sensitivity, thus being poor at excluding malignancy. We found that hypoechogenicity was the most sensitive feature with 54.5% sensitivity compared with 62.7% in the meta-analysis by Remonti et al.^5^

However, incorporating nodule features into clusters and using a recognised system, such as the ATA guidelines, increased the accuracy and probability of a nodule being correctly identified as malignant. This is verified by the high agreement percentage between the assigned ATA classification and cytopathology/histopathology results. This is not only re-assuring for operators performing thyroid sonography, but also for patients, eliminating the need for further invasive testing or surgery. In a resource-constrained setting, such as ours, this alleviates surgical burden, thus freeing up more operating time for cases where surgery is truly indicated.

We found a high agreement between the cytopathology results and the histopathology for patients referred for lobectomies. This highlights the benefit of using a standardised reporting structure such as the Bethesda classification. This also demonstrates a major advantage of performing US-guided FNA; with an increased ability to predict malignancy at surgical resection, many patients are spared the morbidity of unnecessary thyroid surgery.

## Conclusion

This study is the first comprehensive report for Southern Africa correlating sonographic features of thyroid nodules with FNA cytology and histopathology. It confirms that a certain cluster of sonographic features for thyroid nodules aids in predicting thyroid malignancy. Furthermore, the sensitivity and specificity in detecting malignancy, utilising ultrasonography, were consistent with studies performed elsewhere, thus providing reassurance in the usefulness of such a tool, particularly in a resource poor country such as South Africa.

### Limitations of the study

Despite the small sample size, this study significantly contributes to the paucity of data available for sub-Saharan Africa and provides reassurance that our results are consistent with numerous international studies.

Collecting data from a single hospital population limits the applicability of such a study to the racial and socio-economic demographics of Chris Hani Baragwanath Academic Hospital situated in the urban township of Soweto.

Non-diagnostic FNA samples were excluded, thus creating a bias against nodules that were difficult to sample adequately. In addition, analysing combinations of ultrasound features may provide more insight into risk and probability of malignancy rather than individual features alone.

### Recommendations

The findings of this study confirm that the usage of a thyroid nodule classification system improves characterisation and increases accuracy in detecting thyroid nodule malignancies on ultrasound. Thus, it would be prudent for any department performing thyroid sonography to incorporate a classification system into their practice. Furthermore, it would also be useful to compare the two most used thyroid classification systems, ATA and ACR TIRADS, to determine how the two reporting systems compare in terms of their accuracy in detecting malignancy in a thyroid nodule.
